# Diagnostic study of serum claudin-3 and zonulin for intestinal barrier dysfunction after abdominal surgery

**DOI:** 10.3389/fmed.2025.1669630

**Published:** 2025-11-03

**Authors:** YiChen Hu, WenShuo Liu, XueJing Gong, FuQiong Jiang, Jie Huang, YuanPei Zhao, WeiMing Li

**Affiliations:** ^1^Department of Gastrointestinal Surgery, Second Affiliated Hospital of Kunming Medical University, Kunming, China; ^2^Department of Hepatobiliary and General Surgery, Xingtai Central Hospital, Xingtai City, Hebei, China; ^3^Department of General Surgery and Burns, Nujiang Lisu Autonomous Prefecture People’s Hospital, Nujiang, Yunnan, China; ^4^Department of Dermatology, Second Affiliated Hospital of Kunming Medical University, Kunming, China; ^5^Department of Hepatobiliary and Pancreatic Surgery, Second Affiliated Hospital of Kunming Medical University, Kunming, China

**Keywords:** zonulin, claudin-3, intestinal ischemia–reperfusion injury, vintestinal barrierfunction, abdominal surgery

## Abstract

**Background:**

Previous studies have shown that serum claudin-3 and zonulin play important roles in reflecting intestinal barrier function. This study aims to explore the diagnostic value of serum claudin-3 and zonulin in assessing intestinal barrier dysfunction following abdominal surgery.

**Methods:**

A rat model of intestinal ischemia–reperfusion (I/R) injury was established, and hematoxylin and eosin (HE) staining was performed to observe the damage to the intestinal mucosa. Pearson correlation analysis was conducted to assess the relationship between changes in serum claudin-3 and zonulin concentrations and the degree of intestinal mucosal injury. Subsequently, patients undergoing abdominal surgery were included in the clinical study to measure the levels of claudin-3 and zonulin in the blood; peripheral blood *Escherichia coli* 16sDNA was detected using qPCR technology. The diagnostic value of serum claudin-3 and zonulin in relation to intestinal barrier dysfunction was analyzed using ROC curves.

**Results:**

The degree of intestinal mucosal injury in rats and the concentrations of serum claudin-3 and zonulin both exhibited a trend of initially worsening and then improving, peaking at 24 h post-reperfusion. The changes in serum claudin-3 and zonulin concentrations were significantly positively correlated with the degree of intestinal mucosal tissue injury. In patients with intestinal barrier dysfunction, the copy number of *Escherichia coli* and the concentrations of serum claudin-3 and zonulin significantly increased at 24 h post-operation; the area under the ROC curve for serum claudin-3 and zonulin were AUC = 0.934 and AUC = 0.826, respectively.

**Conclusion:**

Following intestinal mucosal injury, the greater the degree of damage, the higher the concentrations of serum claudin-3 and zonulin, which are positively correlated, peaking at 24 h post-operation. Serum claudin-3 and zonulin have good predictive value for postoperative intestinal barrier dysfunction, with serum claudin-3 demonstrating a higher diagnostic value than serum zonulin for postoperative intestinal barrier dysfunction.

## 1 Introduction

The intestinal barrier consists of mechanical, chemical, immune, and biological barriers that collectively defend against the invasion of foreign antigens and maintain homeostasis (1, 2). The mechanical barrier is the most crucial component, primarily composed of tight junctions (TJs), adherens junctions, and desmosomes, with TJs playing a key role (3). TJs are formed by proteins such as claudin, occludin, Zonula Occludens-1 (ZO-1), and zonulin (4, 5). When the body is stimulated, these proteins are disrupted, leading to increased intestinal permeability, which allows harmful substances such as intestinal bacteria and endotoxins to translocate, potentially triggering serious complications such as multiple organ dysfunction syndrome (MODS) or systemic inflammatory response syndrome (SIRS) (6–9). Therefore, early assessment of intestinal barrier function is particularly important. Our research group has previously found that intestinal fatty acid binding protein (I-FABP), D-lactic acid, and citrulline can reflect changes in intestinal barrier function to some extent; however, their testing efficacy is not ideal (10–14). Thus, there is still a need to explore a more convenient biomarker with high sensitivity and specificity to alert against intestinal barrier dysfunction.

Claudin-3 is one of the most important tight junction (TJ) proteins in the intestine, playing a crucial role in controlling intestinal barrier function and mucosal homeostasis. Relevant studies have shown that claudin-3 is highly expressed in the colon and small intestine, making it a promising biomarker for assessing intestinal barrier function (15).

Zonulin, as a mediator that can reversibly regulate intestinal permeability by modulating intercellular tight junctions, plays a significant role in the regulation of intestinal permeability (16). Numerous studies have indicated that zonulin can modulate tight junctions between intestinal epithelial cells, closely linking it to various systems including the digestive system (17–20), central nervous system (21–23), and autoimmune system (24–26).

This study first explores the correlation between serum levels of claudin-3 and zonulin and intestinal barrier dysfunction in rats through animal experiments. Based on this, further clinical research will be conducted to investigate the relationship between serum levels of claudin-3 and zonulin and intestinal barrier impairment in patients post-abdominal surgery, assessing their diagnostic efficacy for intestinal barrier dysfunction.

## 2 Materials and methods

### 2.1 Construction of the rat intestinal ischemia–reperfusion model

A total of 48 male rats, aged 6–8 weeks and weighing between 180 and 220 g, were randomly selected from a Specific Pathogen Free (SPF) environment. These 48 SPF-grade Sprague–Dawley (SD) rats were divided into two groups: a sham operation (Sham) group and an intestinal ischemia–reperfusion (I/R) group, with 24 rats in each group. Subsequently, each group was further randomized into four subgroups based on different reperfusion time points (6 h, 24 h, 48 h, and 72 h), with 6 rats in each subgroup.

All rats were fasted for 12 h prior to surgery. A 10% solution of chloral hydrate was administered intraperitoneally at a dosage of 0.3 ml/100 g, and after preparing the abdomen, the skin was disinfected. A midline incision of approximately 4 cm was made in the abdomen, and the abdominal cavity was exposed layer by layer. The small intestine was exteriorized, and sterile gauze soaked in saline was used to protect the intestinal tissue. The mesentery of the small intestine was fan-shaped and spread out to expose the right abdominal cavity, where the superior mesenteric artery was located. The surrounding tissue was dissected away, and a sterile vascular clamp was applied at the root of the superior mesenteric artery. (In the sham group, after spreading the mesentery of the small intestine and isolating the superior mesenteric artery, no clamping was performed. The intestinal tube was carefully returned to the abdominal cavity, and the abdomen was closed.) After clamping the superior mesenteric artery, the intestinal tissue visibly changed to a pale color, indicating successful ischemia. Sterile gauze moistened with saline was placed over the rat’s abdomen to minimize moisture loss. After 40 min of ischemia, the intestinal tissue changed from pale to dark red. Upon removing the arterial clamp, the intestinal tissue color changed from dark red to bright red, indicating successful reperfusion. The intestinal tube was returned, and the abdominal incision of the rat was sutured. The vital signs of the rats were closely monitored postoperatively. Blood samples were collected from the tail vein of all rats before I/R at 6 h, 24 h, 48 h, and 72 h, and again at the reperfusion time point before euthanizing them by dislocating the cervical vertebrae. Blood was centrifuged at 3500 rpm for 10 min to collect serum, which was stored at −80°C for further analysis. A segment of the intestine approximately 5 cm long was taken 6 cm from the cecum, filled with saline using a syringe to remove intestinal contents, and then fixed in 4% paraformaldehyde for preservation.

### 2.2 H&E staining

The organizational samples were fixed in 4% paraformaldehyde. After deparaffinization and rehydration, they were stained with hematoxylin (Solarbio) for 5 min, followed by eosin (Beyotime) for 3 s. Subsequently, the sections underwent dehydration, clearing, and mounting, and were then observed under a microscope. After examining the rat intestinal tissue sections, scoring was performed using the Chiu’s six-grade scoring system (27), as follows: 0 points: normal intestinal tissue; 1 point: increased subepithelial spaces in intestinal tissue; 2 points: separation of the epithelial layer and the lamina propria; 3 points: separation of the epithelial layer from the lamina propria with concomitant loss of villous tips; 4 points: villous loss with increased inflammatory cells in the lamina propria and capillary dilation; 5 points: destruction of the lamina propria accompanied by hemorrhage and necrosis.

### 2.3 Object of study

This study has been reviewed by the Medical Ethics Committee of the Second Affiliated Hospital of Kunming Medical University (Ethics Approval No. PJ-2020-157). The subjects of the study are patients from the Second Ward of the Gastrointestinal Surgery Department of the Second Affiliated Hospital of Kunming Medical University who met the inclusion criteria from March 2023 to September 2023. All included patients signed informed consent forms.

### 2.4 The levels of serum zonulin and claudin-3 were measured by enzyme-linked immunosorbent assay (ELISA)

The levels of zonulin and claudin-3 in serum were measured using the Enzyme-Linked Immunosorbent Assay (ELISA). During the experiment, the reaction plate was taken out and the wells were set according to the instructions, with sample diluent (blank control), different concentrations of standards, and serum samples to be tested added to each well. Except for the blank wells, a detection antibody labeled with horseradish peroxidase (HRP) was added to each well and incubated in the dark at 37°C for 60 min. After incubation, the liquid was discarded, and the wells were washed repeatedly using a plate washer. Subsequently, a substrate solution was added, and incubation continued in the dark at 37°C for another 60 min. Finally, a stop solution was added to terminate the reaction, and the optical density (OD) values of each well were measured using a microplate reader. The concentrations of zonulin and claudin-3 in the samples were calculated.

### 2.5 Serum zonulin and claudin-3 inclusion criteria and exclusion criteria

#### 2.5.1 Inclusion criteria

(1) Male or female individuals aged between 18 and 75 years; (2) Body mass index (BMI) ranging from 20 to 28 kg/m^2^; (3) Patients who have undergone abdominal surgeries, including radical gastrectomy for gastric cancer, radical resection for colorectal cancer, pancreaticoduodenectomy, cholecystectomy, and appendectomy.

#### 2.5.2 Exclusion criteria

(1) Patients with inflammatory bowel disease; (2) Individuals with a history of medication usage in the past 2 weeks (such as antibiotics, hormones, psychotropic drugs, non-steroidal anti-inflammatory drugs, etc.); (3) Individuals with a history of abdominal surgery in the past month or those who experienced acute bleeding, obstruction, or perforation prior to surgery; (4) Tumor patients who received neoadjuvant radiochemotherapy, targeted therapy, or immunotherapy prior to surgery; (5) Individuals with autoimmune diseases; (6) Patients with diabetes.

### 2.6 Evaluation indicators of intestinal barrier dysfunction

According to the 2006 clinical diagnosis and treatment recommendations for intestinal barrier dysfunction by the Gastroenterology Branch of the Chinese Medical Association (28): (1) Patients present with critical illnesses that may lead to intestinal barrier dysfunction; (2) Symptoms such as abdominal pain, bloating, diarrhea, or constipation, as well as gastrointestinal bleeding, inability to tolerate food, and signs of reduced or absent bowel sounds (excluding changes in bowel sounds due to anesthesia or medication) appear on the basis of the primary disease. Furthermore, the concentration of *Escherichia coli* (*E. coli*) in the blood is assessed using quantitative polymerase chain reaction (qPCR) technology to evaluate the presence of microbial translocation, thereby determining whether the patient has intestinal barrier dysfunction.

### 2.7 Peripheral blood *Escherichia coli* 16SrDNA qPCR detection

To achieve quantitative detection of *Escherichia coli* 16S rDNA in peripheral blood, *E. coli* was first cultured through amplification, and its optical density (OD600) was measured during the logarithmic growth phase to calculate the concentration of the bacterial suspension. Subsequently, plasmid DNA was extracted and its purity and integrity were verified through agarose gel electrophoresis. The obtained plasmid was used as a standard, and after a series of gradient dilutions, it was employed to construct a standard curve. Meanwhile, a commercial DNA extraction kit was used to extract total DNA from the patient’s peripheral blood, and the quality of the DNA was validated through electrophoresis. During the quantitative detection process, TProbe qPCR Mix and TaqMan specific probes were used for real-time fluorescent quantitative PCR reactions. Based on the amplification results of the standard plasmid with known copy numbers, the corresponding copy numbers for each concentration were calculated, and a standard curve of log copy numbers versus cycle threshold (CT values) was plotted. Finally, the CT values obtained from clinical blood samples were substituted into the standard curve for conversion, yielding the absolute copy numbers of *E. coli* 16S rDNA in peripheral blood, thus enabling quantitative assessment of bacterial load in the blood.

### 2.8 Statistical calculation

Statistical analysis of the data was performed using SPSS 26.0 software. For normally distributed continuous data, results are expressed as mean ± standard deviation (mean ± SD). For non-normally distributed data, results are expressed as median and interquartile range. For normally distributed data, the independent samples t-test was used; for non-normally distributed data, the rank-sum test was employed. Comparisons between groups were conducted using analysis of variance (ANOVA). The ROC curve was utilized to analyze the correlation and diagnostic value of serum claudin-3 and zonulin with intestinal barrier dysfunction. A *p*-value of <0.05 was considered statistically significant.

## 3 Results

### 3.1 Histopathological changes of intestinal mucosa in rats after I/R injury

**Figure 1:**
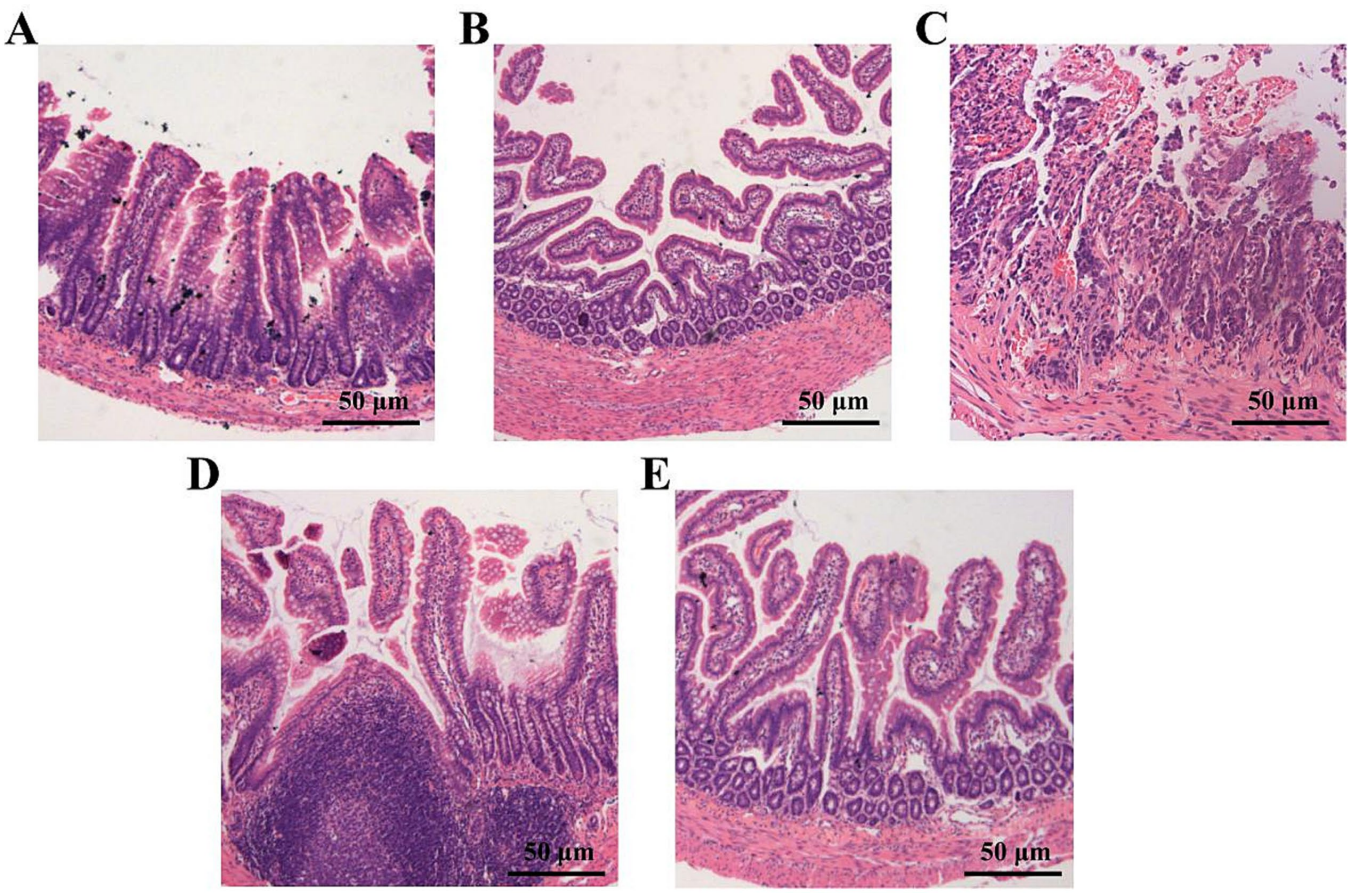
Representative pathological changes in the intestinal mucosa of the sham operation group and the intestinal ischemia–reperfusion (I/R) group at different time points (HE × 50). **(A)** Sham operation group: The structure of intestinal villi is intact, and the epithelium is continuous. **(B)** I/R 6 h: There is partial separation between the epithelium and the lamina propria, with some villus tips shedding. **(C)** I/R 24 h: The mucosal damage is most severe, characterized by extensive shedding of villi, accompanied by bleeding and necrosis. **(D)** I/R 48 h: Some villi have shed, and there is significant hyperplasia of lymphoid follicles. **(E)** I/R 72 h: The structure of the intestinal mucosa gradually recovers, but an enlarged subepithelial space is still observable.

Under the microscope, observation of rat intestinal tissue reveals that in the sham, the intestinal mucosal structure is intact, with villi arranged in a regular pattern (Figure 1A). In the I/R 6 h group, separation occurs between the lamina propria and the epithelial layer, with some villi tips shedding (Figure 1B). The I/R 24 h group shows shedding of villi and the lamina propria, accompanied by hemorrhage and necrosis (Figure 1C). In the I/R 48 h group, partial shedding of villi is observed, with significant lymphoid follicle hyperplasia (Figure 1D). The I/R 72 h group shows that the intestinal mucosal tissue begins to gradually return to normal, although an increase in the subepithelial space is still evident (Figure 1E).

### 3.2 Chiu’s histological scoring reveals peak intestinal mucosal damage at 24 h post-ischemia–reperfusion in rats

After observing and statistically analyzing the intestinal tissue of rats (Table 1), it was found that the degree of intestinal injury began to worsen at 6 h post-reperfusion, reaching a peak at 24 h, and subsequently began to gradually alleviate. By 72 h, the condition was trending towards normal, but still remained higher than that of the sham-operated group (*p* < 0.05).

**Table 1:** Intestinal tissue injury scores at different time points and comparisons.

Score	Grouping					F	P
Chiu’s score	Sham	I/R6h	I/R24h	I/R48h	I/R72h	54.92	0
	0.21 ± 0.51	2.67 ± 0.81	4 ± 0.89	2.83 ± 0.75	1.67 ± 0.75		

I/R, ischemia–reperfusion.

### 3.3 Dynamic changes and group comparisons of serum zonulin/claudin-3 concentrations in rats after ischemia–reperfusion

**Figure 2:**
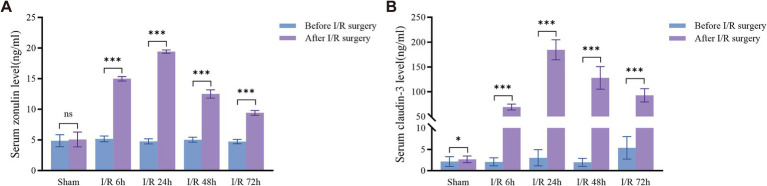
Dynamic changes of serum zonulin and claudin-3 concentrations after I/R injury in rats. **(A,B)** Compared with the sham group, serum zonulin and claudin-3 levels significantly increased at different time points after I/R (6, 24, 48, and 72 h), peaking at 24 h post-reperfusion and then gradually declining, but remaining higher than those of the sham group. *p < 0.05; **p < 0.01; ***p < 0.001. ns, not significant.

Compared to the preoperative and sham groups, the serum concentration of zonulin in the intestinal ischemia–reperfusion group of rats began to rise at I/R 6 h, peaked at I/R 24 h, and then gradually decreased, although it remained higher than that of the sham group (Figure 2A). Compared to the preoperative and sham groups, the serum concentration of claudin-3 in the intestinal ischemia–reperfusion group of rats began to rise at I/R 6 h, peaked at I/R 24 h, and then gradually decreased, although it remained higher than that of the sham group (Figure 2B).

### 3.4 Correlation between serum zonulin/claudin-3 levels and histological severity scores of intestinal injury

**Figure 3:**
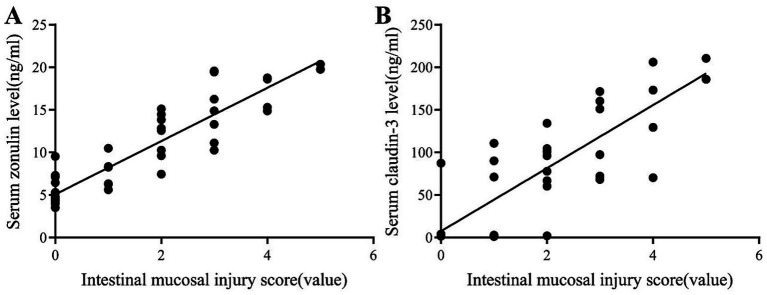
Analysis of the correlation between serum biomarkers and histological damage scores after intestinal ischemia–reperfusion in rats. **(A)** The serum zonulin level showed a significant positive correlation with Chiu’s histological score (r = 0.916, *p* < 0.001). **(B)** The serum claudin-3 level also exhibited a significant positive correlation with the histological damage score (r = 0.888, *p* < 0.001).

The expression level of serum zonulin is significantly positively correlated with the degree of intestinal tissue damage (r = 0.916, *p* < 0.001), and the serum claudin-3 level also shows a positive correlation with the rating of intestinal mucosal injury (r = 0.888, *p* < 0.001) (Figures 3A,B).

### 3.5 Clinical sample characteristics of patients undergoing abdominal surgery

A total of 43 patients who underwent abdominal surgery at our hospital were included in this study, which comprised 20 cases of colorectal cancer patients undergoing radical resection for colorectal cancer, 7 cases of pancreaticoduodenectomy, 7 cases of radical resection for gastric cancer, 6 cases of appendectomy, and 3 cases of cholecystectomy (Table 2).

**Table 2:** The basic characteristics of the research subjects.

Case characteristics	Number of cases
Gender (n, male/female)	26/17
Age (mean ± SD)	66.02 ± 1.26
Types of diseases	
radical resection for colorectal cancer	20
Pancreaticoduodenectomy	7
Radical resection for gastric cancer	7
Appendicectomy	6
Cholecystectomy	3

SD, standard deviation.

### 3.6 Clinical evaluation of postoperative intestinal barrier dysfunction in patients

The concentration of *Escherichia coli* in peripheral blood was detected by qPCR, and combined with the assessment of the patients’ clinical characteristics, it was found that 19 patients experienced intestinal barrier dysfunction on the first postoperative day.

### 3.7 Peripheral blood *Escherichia coli* 16S rDNA detection and comparative analysis between patient groups

**Figure 4:**
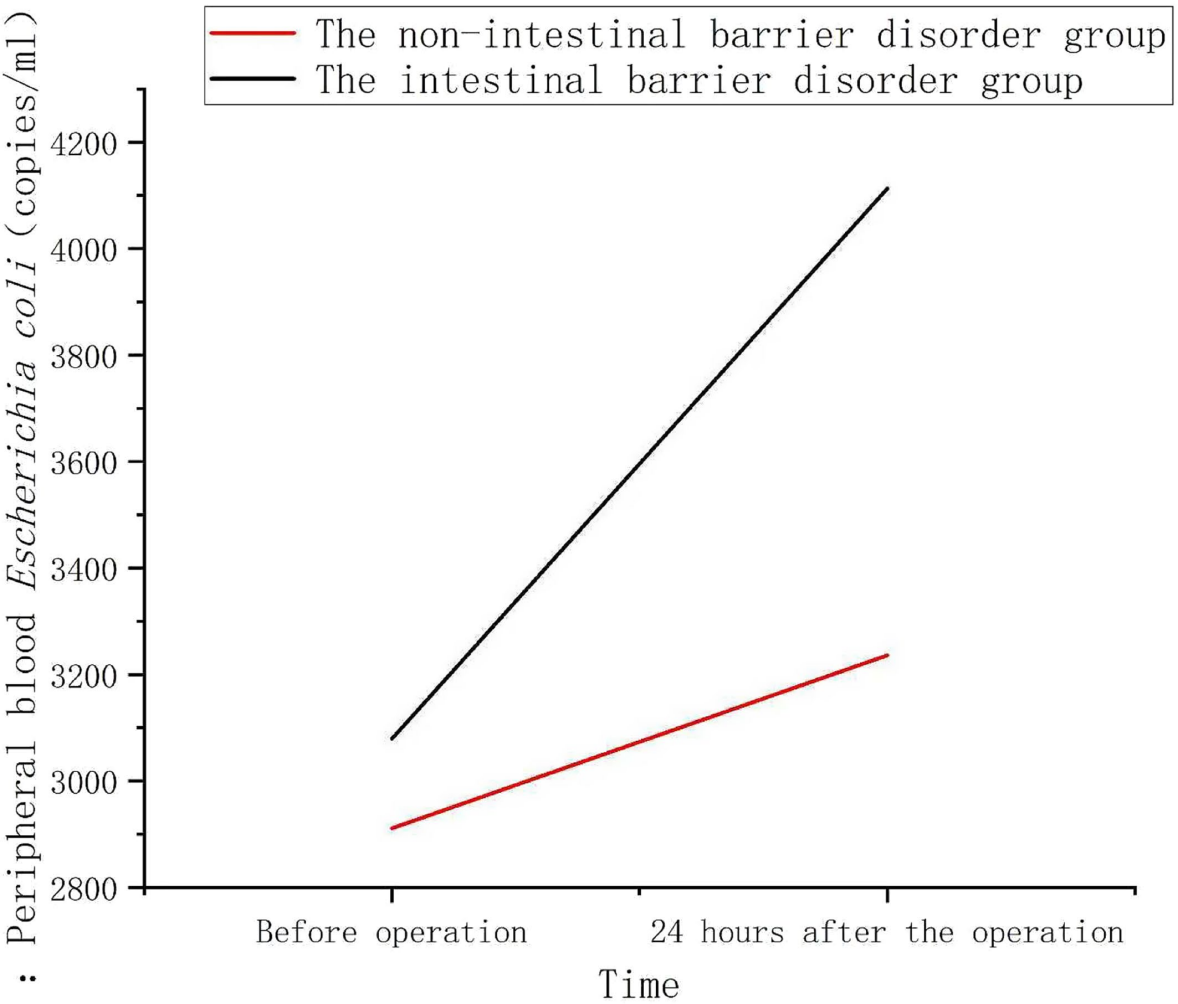
Changes of peripheral blood *Escherichia coli* 16S rDNA levels in patients with or without intestinal barrier dysfunction. There was no significant difference in the copy number of *E. coli* 16S rDNA in the peripheral blood of the two groups of patients before surgery. Twenty-four hours after surgery, the levels in both groups increased, with the intestinal barrier dysfunction group showing a more significant increase, which was markedly higher than that of the non-dysfunction group (*p* < 0.001).

In the non-intestinal barrier impairment group, the increase in peripheral blood *E. coli* copy numbers at 24 h post-operation was relatively low. In contrast, in the intestinal barrier impairment group, the increase in peripheral blood *Escherichia coli* copy numbers at 24 h post-operation was significantly higher (Figure 4).

### 3.8 Comparative analysis of serum zonulin and claudin-3 levels in patients with and without intestinal barrier dysfunction

In the non-intestinal barrier impairment group, the serum claudin-3 concentration at 24 h post-operation was increased to a lesser extent (Table 3); in the intestinal barrier impairment group, the serum claudin-3 concentration at 24 h post-operation was significantly elevated (Table 4). Compared with the non-intestinal barrier impairment group, the serum claudin-3 concentration at 24 h post-operation in the intestinal barrier impairment group was significantly higher (Table 5). Additionally, compared to the non-intestinal barrier impairment group, the serum zonulin concentration at 24 h post-operation in the intestinal barrier impairment group showed a marked increase (Table 5).

**Table 3:** Serum concentrations of zonulin and claudin-3 in the non-intestinal barrier disorder group.

The non-intestinal barrier disorder group	Before operation	24 h after the operation	T	*p*
Serum zonulin (ng/ml)	5.6792 ± 1.44	13.2425 ± 2.52	−12.177	<0.001
Serum claudin-3 (ng/ml)	0.80604 ± 0.62	1.74283 ± 1.31	−3.826	0.001

SD, standard deviation.

**Table 4:** Serum concentrations of zonulin and claudin-3 in the intestinal barrier disorder group.

The intestinal barrier disorder group	Before operation	24 h after the operation	T	*p*
Serum zonulin (ng/ml)	6.2379 ± 1.235	16.3668 ± 2.166	−18.108	<0.001
Serum claudin-3 (ng/ml)	1.70553 ± 0.8088	5.21721 ± 1.711	−18.108	<0.001

SD, standard deviation.

**Table 5:** Comparison of serum zonulin and claudin-3 concentrations 24 h after surgery between the non-intestinal barrier dysfunction group and the intestinal barrier dysfunction group.

24 h after the operation	The non-intestinal barrier disorder group	The intestinal barrier disorder group	T	*p*
Serum zonulin (ng/ml)	13.2425 ± 2.526	16.3668 ± 2.166	4.284	<0.001
Serum claudin-3 (ng/ml)	1.74283 ± 1.310	5.21721 ± 1.711	7.546	<0.001

SD, standard deviation.

### 3.9 Diagnostic performance of serum zonulin and claudin-3 in predicting postoperative intestinal barrier dysfunction

**Figure 5:**
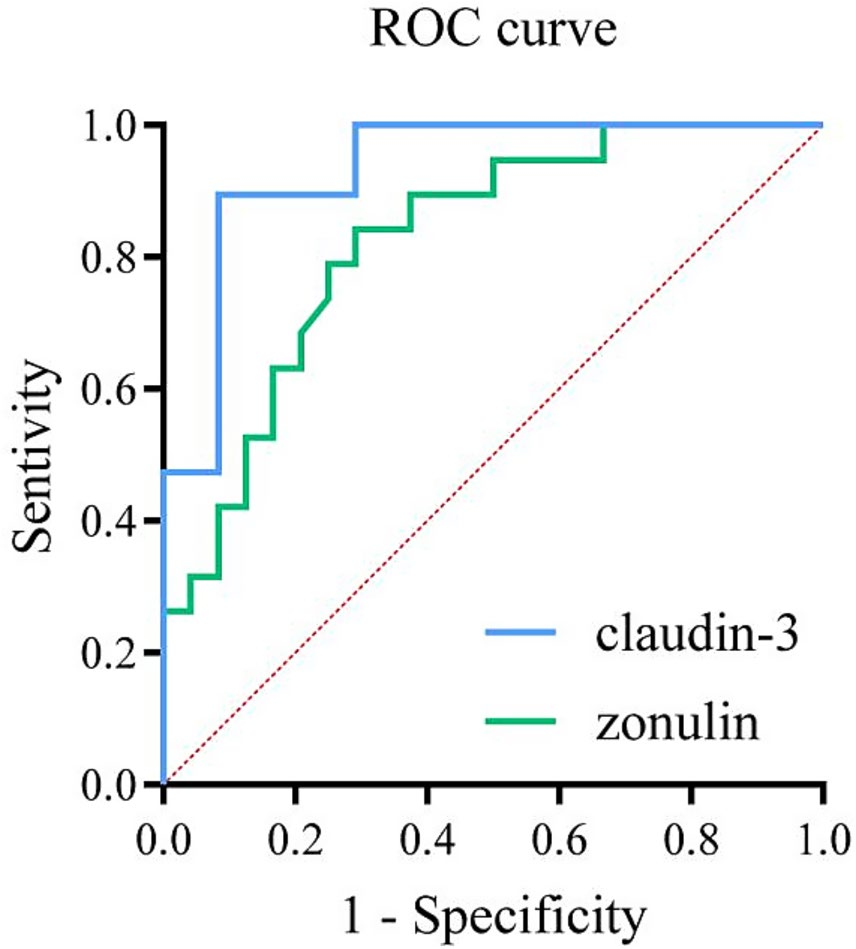
ROC curve analysis of serum zonulin and claudin-3 in diagnosing intestinal barrier dysfunction on the first postoperative day in patients undergoing abdominal surgery. The area under the curve (AUC) for serum zonulin was 0.826 (optimal cutoff value 14.35 ng/ml; sensitivity 84.2%; specificity 70.8%), while the AUC for serum claudin-3 was 0.934 (optimal cutoff value 3.253 ng/ml; sensitivity 89.5%; specificity 91.7%).

The ROC curve was plotted to evaluate the diagnostic efficacy of serum zonulin on the first postoperative day for intestinal barrier dysfunction, resulting in an area under the curve (AUC) of 0.826 (*p* < 0.001). The optimal cutoff value for predicting intestinal barrier dysfunction was found to be 14.355 ng/ml. Additionally, the ROC curve analysis for serum claudin-3 on the first postoperative day yielded an AUC of 0.934 (*p* < 0.001), with the optimal cutoff value for predicting intestinal barrier dysfunction determined to be 3.253 ng/ml (Figure 5; Table 6).

**Table 6:** The diagnostic efficacy of serum claudin-3 and zonulin 24 h after the operation.

24 h after the operation	Critical value (ng/ml)	Sensitivity	Specificity	AUC
Claudin-3	3.253	0.895	0.917	0.934
Zonulin	14.355	0.842	0.708	0.826

AUC, area under the curve.

## 4 Discussion

According to previous studies, the diagnostic value of serum biomarkers such as I-FABP, D-lactate, and citrulline for intestinal barrier dysfunction has been investigated, with D-lactate showing the highest diagnostic value (AUC = 0.84) (29, 30). Subsequently, a multi-marker combined diagnostic approach was employed, which demonstrated a higher overall diagnostic value compared to individual markers; however, its specificity was not ideal. Therefore, there is a need to explore a more convenient biomarker with high sensitivity and specificity to alert for intestinal barrier dysfunction.

Previous research on zonulin has primarily focused on chronic diseases such as celiac disease, irritable bowel syndrome, and inflammatory bowel disease. Celiac disease (CD), as an autoimmune intestinal disorder, has provided the majority of our understanding of zonulin through studies conducted on CD patients. CD has been used as a model disease to investigate the role of zonulin (31). The disruption of tight junction structures driven by zonulin plays a crucial role in the progression of celiac disease (19). Studies have shown that zonulin levels are elevated in the tissues and serum of CD patients, and as zonulin levels increase, the clinical symptoms of patients also worsen (20, 32). In patients with inflammatory bowel disease (IBD), levels of zonulin in serum and feces are significantly elevated, particularly during active phases, showing a clear positive correlation between intestinal permeability and zonulin levels. Additionally, serum zonulin levels in CD patients are higher than those in ulcerative colitis (UC) patients (33). Ling et al. confirmed through *in vitro* cell experiments that zonulin participates in the pathogenesis of necrotizing enterocolitis by regulating intestinal permeability, and treatment with Bifidobacterium can mitigate intestinal permeability and reduce serum zonulin levels (34). Diana et al. found a significant positive correlation between elevated zonulin levels and increased lipopolysaccharide levels in neonates (35). Diarrhea-predominant irritable bowel syndrome (IBS) is a common functional gastrointestinal disorder. Some scholars have found that the levels of zonulin in the serum and feces of IBS patients are elevated compared to healthy individuals, and this concentration is positively correlated with the severity of diarrhea (36). This provides substantial evidence that zonulin is significantly associated with intestinal permeability.

Claudin-3 is one of the most abundantly expressed claudin proteins in the intestine, primarily localized in the distal gastrointestinal tract, where it forms tight junctions between the epithelial cells of the ileum and colon. When these tight junctions are disrupted, intestinal permeability increases, and the protein sheds from the intestinal epithelial cells into the bloodstream, leading to elevated concentrations in peripheral blood (37, 38). Studies have shown a positive correlation between the expression of claudin-3 and the maturity and integrity of the intestinal barrier (39). An animal experiment indicated that the levels of claudin-3 within intestinal tissues increase with the maturation of the intestine and the enhancement of barrier integrity; a decrease in its content leads to increased intestinal permeability (40), further confirming the close relationship between claudin-3 and the intestinal barrier.

In this study, we utilized a rat model of intestinal ischemia–reperfusion to investigate the relationship between the dynamic changes in serum zonulin and claudin-3 and intestinal mucosal injury. The results indicated that the degree of intestinal mucosal injury in the ischemia–reperfusion group was significantly correlated with the concentrations of serum zonulin and claudin-3, which increased significantly with prolonged ischemia–reperfusion time, peaking at 24 h post-reperfusion and then gradually decreasing, yet remaining higher than that of the sham-operated group. Correlation analysis revealed a significant positive correlation between serum zonulin and claudin-3 expression and the degree of intestinal tissue injury. This experiment preliminarily demonstrated a positive correlation between the changes in serum zonulin and claudin-3 expression and the degree of intestinal mucosal injury, suggesting that their concentration changes could reflect the condition of intestinal mucosal barrier damage to some extent, providing a basis for further clinical research.

To further investigate whether the concentrations of serum zonulin and claudin-3 after surgical trauma can be used to diagnose impaired intestinal barrier function, we included 43 patients who underwent abdominal surgery at our hospital. Referring to previous studies (30), we utilized qPCR technology to detect the peripheral blood levels of *Escherichia coli* 16S rDNA at different postoperative time points. Combined with clinical manifestations, we assessed whether the patients exhibited intestinal barrier dysfunction within 24 h post-surgery, categorizing them into an intestinal barrier dysfunction group and a non-intestinal barrier dysfunction group. We then analyzed the differences in serum zonulin and claudin-3 concentrations between the two groups and conducted ROC curve analysis to evaluate their diagnostic value for intestinal barrier dysfunction in patients after abdominal surgery.

In patients with intestinal barrier dysfunction, the serum concentrations of zonulin and claudin-3 at 24 h post-operation were significantly higher than those before surgery, and the increase was similarly pronounced compared to the non-intestinal barrier dysfunction group. ROC curve analysis of serum zonulin and claudin-3 on the first postoperative day in relation to the occurrence of intestinal barrier dysfunction revealed that serum zonulin had a diagnostic efficacy with an AUC of 0.826, a best cutoff value of 14.35 ng/ml, sensitivity of 0.842, and specificity of 0.708, indicating that serum zonulin has a good early warning value for intestinal barrier dysfunction. In contrast, serum claudin-3 demonstrated a diagnostic efficacy with an AUC of 0.934, a best cutoff value of 3.253 ng/ml, sensitivity of 0.895, and specificity of 0.917, suggesting that serum claudin-3 has a very good early warning value for intestinal barrier dysfunction and possesses higher diagnostic value than serum zonulin.

Claudin-3 is a core member of tight junction proteins and is directly involved in maintaining the structural integrity of the epithelial barrier. Therefore, during ischemia–reperfusion injury or surgical trauma, its serum levels can more directly and sensitively reflect the degree of tight junction disruption (39). This may explain why Claudin-3 exhibited a higher AUC value (0.934) in the ROC curve analysis of this study, demonstrating good sensitivity and specificity. In contrast, Zonulin primarily affects barrier permeability by reversibly regulating tight junction opening. Due to its susceptibility to various inflammatory and metabolic factors, its specificity is relatively low (20), which resonates with the findings of this study.

Further literature indicates that elevated levels of Claudin-3 often signify the disruption of tight junction structures, suggesting a higher degree of irreversibility of damage. For instance, in studies involving patients with inflammatory bowel disease, the downregulation of Claudin-3 is highly correlated with mucosal barrier disruption and microbial translocation, while increased peripheral blood concentrations are believed to reflect severe impairment of intestinal epithelial integrity (41). In the rat model of this study, the trend of serum levels of Claudin-3 was completely consistent with histological scoring, further validating its value as a direct indicator of structural damage. In contrast, elevated levels of Zonulin more often indicate functional barrier relaxation, a change that can be reversible in certain circumstances; for example, probiotics, nutritional interventions, or immunomodulatory treatments can reduce Zonulin levels and improve intestinal permeability (42). Therefore, Claudin-3 is more suitable as a diagnostic indicator, while Zonulin may have advantages in monitoring treatment effects or early warning.

It is noteworthy that intestinal barrier damage is not only a localized issue but is also closely related to systemic complications. Existing studies have shown that barrier dysfunction can lead to bacterial translocation and endotoxemia, triggering the systemic inflammatory response syndrome (SIRS) and multiple organ dysfunction syndrome (MODS) (43). In this study, the copy number of *Escherichia coli* 16S rDNA in the peripheral blood of patients in the barrier damage group was significantly elevated, validating this mechanism. In conjunction with previous research on colorectal cancer surgery, the perioperative use of probiotics can significantly reduce infectious complications, accompanied by a decrease in Zonulin levels (42), suggesting that serum biomarkers can not only be used for diagnosis but also serve as reference indicators for therapeutic efficacy. Therefore, incorporating Claudin-3 and Zonulin into the clinical monitoring system is expected to assist surgeons in early identification of high-risk patients postoperatively, allowing for timely intervention and thereby reducing the incidence of severe complications.

However, this study also has certain limitations. First, the sample size is small and the study is a single-center investigation, which may affect the generalizability of the results. Second, classical permeability tests, such as lactulose/mannitol absorption tests, were not used as controls, resulting in a lack of standardized references. Furthermore, although the elevation of Claudin-3 and Zonulin is closely related to barrier dysfunction, their molecular mechanisms remain unclear, particularly regarding the interactions between the two in different disease contexts, which require further investigation. Studies have shown that the intestinal epithelial repair process involves multiple signaling pathways, including Wnt/β-catenin, Hippo, and Notch pathways (44). However, there is currently a lack of evidence to directly link these pathways with changes in the expression of Claudin-3 or Zonulin. Therefore, future research could integrate multi-omics approaches to further elucidate their molecular mechanisms.

To promote the clinical application of Claudin-3 and Zonulin, multiple efforts are required: First, conducting multicenter, large-sample prospective studies to validate their stability and universality across different types of surgeries and patient populations; Second, exploring a multi-index combined prediction model that integrates Claudin-3, Zonulin, D-lactate, I-FABP, and microbiome characteristics, utilizing machine learning methods to construct a comprehensive predictive tool for postoperative barrier dysfunction; Third, attempting to employ these indicators as primary endpoints in interventional studies, such as probiotics, short-chain fatty acid supplementation, and optimization of enteral nutrition, to assess whether they can improve prognosis by reducing Zonulin or stabilizing Claudin-3 levels; Fourth, extending the observation period to study the dynamic changes of these indicators within days or even weeks after surgery, in order to better depict the entire process of intestinal barrier injury and repair.

In summary, this study confirms the significant role of Claudin-3 and Zonulin in postoperative intestinal barrier dysfunction. Claudin-3 better reflects structural damage, possesses higher specificity, and has superior diagnostic value compared to Zonulin; whereas Zonulin has unique advantages in indicating functional barrier relaxation and monitoring intervention effects. The combined use of both biomarkers not only enhances diagnostic accuracy but may also provide important evidence for perioperative risk stratification and individualized interventions. With the advancement of future research, Claudin-3 and Zonulin are expected to become core serological tools in the clinical management of postoperative intestinal barrier dysfunction, thereby improving overall patient prognosis.

## 5 Conclusion

The expression levels of serum zonulin and claudin-3 exhibited a trend of initial increase followed by a decrease after surgical trauma, peaking at 24 h post-operation. Both serum zonulin and claudin-3 demonstrate significant diagnostic value for postoperative intestinal barrier dysfunction, with serum claudin-3 showing a higher diagnostic value than serum zonulin for postoperative intestinal barrier impairment.

## Data Availability

The datasets presented in this study can be found in online repositories. The names of the repository/repositories and accession number(s) can be found in the article/Supplementary material.
